# Public Health Guidelines for Physical Activity: Is There an App for That? A Review of Android and Apple App Stores

**DOI:** 10.2196/mhealth.4003

**Published:** 2015-05-21

**Authors:** Emily Knight, Melanie I Stuckey, Harry Prapavessis, Robert J Petrella

**Affiliations:** ^1^University of Western OntarioFaculty of Health SciencesLondon, ONCanada; ^2^Aging, Rehabilitation and Geriatric Care Research CentreLawson Health Research InstituteLondon, ONCanada; ^3^Computer ScienceUniversity of Western OntarioLondon, ONCanada; ^4^Schulich School of Medicine and DentistryUniversity of Western OntarioLondon, ONCanada; ^5^University of Western OntarioCentre for Studies in Family MedicineLondon, ONCanada

**Keywords:** Mobile applications, Exercise, Public Health

## Abstract

**Background:**

Physical activity participation is an important behavior for modifying lifestyle-related disease risk. Mobile health apps for chronic disease management and prevention are being developed at a rapid rate. However, it is unclear whether these apps are evidence-based. Current public health recommendations for physical activity participation for adults highlight the importance of engaging in 150 minutes weekly of purposeful exercise, and muscle strengthening activities on at least 2 days of the week.

**Objective:**

The aims of the present review were to (1) identify available evidence-based physical activity apps, and (2) identify technological features that could be leveraged to improve health outcomes.

**Methods:**

iTunes and Google Play mobile app stores were searched using keyword and category searching during a single day (February 18, 2014) for physical activity apps available in English. The description pages of eligible apps were reviewed by 4 independent reviewers for evidence-based content, technological, and descriptive features. An a priori subset of apps was downloaded for further review (n=6 affiliated with a non-commercial agency; n=10 top rated; n=10 random selection), and developers were contacted for information regarding evidence-informed content.

**Results:**

The initial search yielded 2400 apps, of which 379 apps (n=206 iTunes; n=173 Google Play) were eligible. Primary results demonstrated no apps (n=0) adhering to evidence-based guidelines for aerobic physical activity, and 7 out of 379 implementing evidence-based guidelines for resistance training physical activity. Technological features of apps included social networking (n=207), pairing with a peripheral health device (n=61), and measuring additional health parameters (n=139). Secondary results revealed 1 app that referenced physical activity guidelines (150 minutes/weekly of exercise), and demonstrated that apps were based on various physical activity reports (n=4) or personal expertise (n=2).

**Conclusions:**

The present study demonstrated a shortage of evidence-based physical activity apps. This gap underscores the need for development of evidence-informed mobile apps. Results highlight the opportunity to develop evidence-informed mobile apps that can be used clinically to enhance health outcomes.

## Introduction

### Background

**Figure 1:**
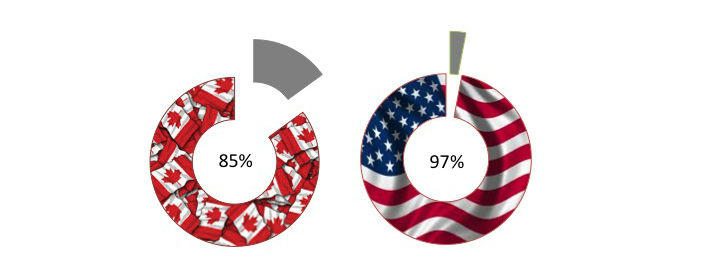
Physical inactivity burden in North America. 85% of Canadian adults and 97% of American adults fail to meet public health guidelines for physical activity.

Health systems worldwide are being increasingly challenged by care for chronic conditions and non-communicable diseases such as cardiovascular disease, cancer, and diabetes [1,2]. In North America, 89% of total mortality in Canada and 87% in the United States can be attributed to non-communicable disease [3]. Physical activity is an important determinant of health, including primary and secondary prevention of chronic and non-communicable diseases [4]. Physically inactive lifestyles are the fourth leading cause of death, and globally contribute to more than 3 million deaths per year [5]. In North America, the majority of adults in the United States and Canada are not meeting minimum public health recommendations for physical activity [6,7]. Engaging in unhealthy physical activity behaviors, such as a physically inactive lifestyle, has substantial negative consequences for public health including the economic burden for society [5] (see Figure 1).

### Physical Activity

Public health guidelines for physical activity are a summary of best available evidence [5,8-10]. For the general adult and older adult population (ie, ≥18 years), physical activity guidelines recommend engaging in at least 150 minutes of moderate- to vigorous-intensity physical activity weekly (ie, aerobic exercise), in bouts of at least 10 minutes, as well as whole body strength training activities for major muscle groups on at least 2 days per week (Textbox 1). Additionally the guidelines highlight that more physical activity is beneficial for health, and that older adults (≥65 years) benefit from balance training to reduce fall risk.

**Evidence-based physical activity guidelines for adults.**

Aerobic Activity

- 150 minutes of moderate- to vigorous-intensity physical activity, accumulated in bouts ≥10 minutes
- More activity is beneficial for health

Strengthening Activity

- Resistance training to strengthen major muscle groups on ≥2 days/week

### Prescribing Physical Activity

Health care providers prescribe treatment regimens to help clients manage their health. A written prescription holds symbolic meaning for clients, indicating that their health practitioner believes in the value of the behavior for managing or promoting health [11]. A health behavior message, such as physical activity, delivered by a health practitioner may be an important stimulus for individual change [12,13]. The evidence base demonstrates the efficacy of prescribing exercise behaviors through primary care for improving both physical activity levels and cardiovascular health [14]. However, the extent to which a client adheres to prescribed behaviors is highly variable, and clinicians may need to consider providing additional tools and interventions for clients to promote adoption of prescribed behaviors [13]. Client self-management and medical technologies can be leveraged to increase engagement in prescribed behaviors by attracting and involving patients in their own care [2,15].

### Mobile Health Apps

The use of mobile health technologies involving smartphones (ie, broadband-enabled phones with the capacity to download and run apps) is a rapidly growing focus for chronic disease management and prevention [16]. Around the world, there are more than two billion smartphone subscriptions [17]. Moreover, in 2014 there was an increase of 400 million subscriptions from the previous year [17]. In North America, 95% of Americans and 80% of Canadians have active smartphone subscriptions [17]. Mobile health technologies have demonstrated potential for engaging individuals in on-going self-management of prescribed physical activity behaviors for disease management and prevention [16,18-20]. It has been suggested that mobile health devices have the ability to deliver multifaceted behavior change interventions using health apps [21]. Moreover, technology-enhanced features included in apps have the potential to reduce user burden and facilitate behavior change through features such as integrated measurement of additional health parameters, social networking, reminders to engage in a behavior, calendar for scheduling behavior, and prompts for lapses in adherence with behaviors [22]. Smartphone subscriptions are pervasive around the world, and leveraging the accessibility of mobile health apps could hold promise for health practitioners and health behavior intervention.

Physical activity apps are abundant. On any given day, a category search (eg, “Health & Fitness”) or keyword search (eg, “physical activity”) of any platform will generate thousands of search results. A recent systematic review reported the use of apps to increase physical activity [23]. However, the authors noted that few apps have been examined through rigorous intervention [23], which underscores the challenge for users to select an evidence-based app. The challenge for clients and health practitioners is discerning which apps, if any, to use to promote prescribed health behaviors. Previous studies have explored the availability of mobile phone apps to change smoking behaviors, manage diabetes, and enhance weight loss outcomes [21,22,24]. These studies showed that available apps were generally not evidence-based, though some evidence-based features were included. While previous research has concluded that physical activity apps lack sufficient inclusion of evidenced-based health behavior change theories [25], it remains unclear whether available physical activity apps contain evidence-based physical activity content and could be used by health practitioners to counsel patients on healthy physical activity behaviors.

### Purpose

The aim of the present review was to identify publically available mobile physical activity apps that represent the evidence-based public health guidelines for physical activity (Textbox 1). Additionally, technological features of apps that have previously been shown to improve utility as well as promote adherence and health outcomes [22] were identified.

## Methods

### Search Process

The search strategy was developed based on previously published studies examining evidence-based apps [22,24]. The mobile app stores for Apple (iTunes) and Android (Google Play) platforms were searched in February 2014 by 4 independent reviewers (2 per platform). Keyword (“physical activity”, “fitness”, “walking”, and “pedometer”) and category (“Health & Fitness” both free and paid) searching was conducted during a single day (February 18, 2014) to determine available apps. The search optimization was set to “relevance”, meaning that results were presented in descending order of relevance using app store algorithms.

### Primary Review

Building on the methods reported by Breton et al [24], the app description pages provided by each app store (iTunes and Google Play) of the first 100 results from each search (ie, 6 searches x 2 platforms) were screened for eligibility. Eligibility criteria included availability in English (demonstrated through text and/or screenshots provided in the description), primary aim of app was physical activity (eg, diet apps with secondary option to track physical activity, sleep tracking apps, or menstrual cycle tracking apps were ineligible), and the app tracked or measured physical activity. Data were extracted from the description page for all eligible apps, which included descriptive data for physical activity content, social behavior (eg, linking with social networks), and clinical utility (eg, cost, linking with peripheral health devices), as well as user ratings and number of downloads (available for Google Play only). While this search strategy is limited to information available on the description page, it represents the typical process a user (clinician or client) would follow when selecting an app for download.

### Assessing Evidence-Based Content

The eligible apps were compared to physical activity guidelines to assess for evidence-based content. Specifically, app descriptions that included 150 minutes/weekly of moderate- to vigorous-intensity physical activity or ≥2 days/weekly of whole body strengthening activities were considered to reflect evidence-based guidelines. Adherence to public health recommendations for physical activity was coded as present/absent, and detailed description was added if present.

### Assessing App Features

Features that could be important in designing future apps or in assisting clinicians and clients in selecting an app for use were identified. An a priori list of general and technological features was created to assist the review process. General features of eligible apps were recorded, including platform, cost, and user ratings. It was also noted if the app was endorsed or affiliated with an agency (eg, government, academic, commercial). Description of how physical activity was measured within the app, as well as any additional health parameters that were tracked using the app were recorded. Technological features of the app were also recorded, including capacity for reminders, calendars/scheduling, social networking, and connecting with other peripheral devices (eg, health devices, computers).

### Secondary Review

Building on the methods of Pagoto et al [22], a subset of apps were downloaded for further review: (1) apps that were endorsed by a non-commercial agency (eg, a university or research group), (2) random selection of top-rated apps (ie, user ratings above 4.5 “stars”), and (3) random selection of remaining apps. Random selection was conducted using a Web-based randomization tool [26]. Additionally, publishers of the subset of downloaded apps were contacted via the email provided on the app’s description page to inquire about the physical activity evidence base that informed the app’s development process.

### Statistical Methods

Descriptive statistics were used to summarize the features available among all eligible apps. Data was managed in Microsoft Excel for Mac 2011. Data were collected in February 2014, and analyzed in March 2014.

## Results

### Overview

**Figure 2:**
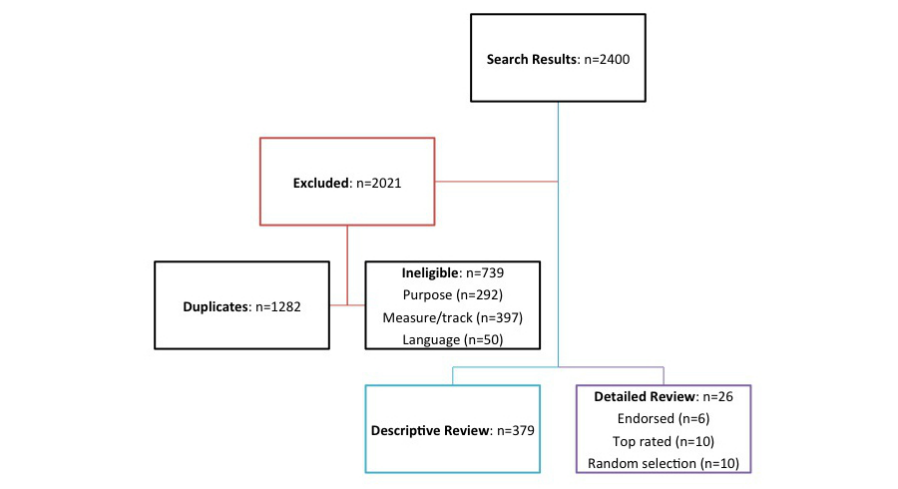
Flow of search results.

**Figure 3:**
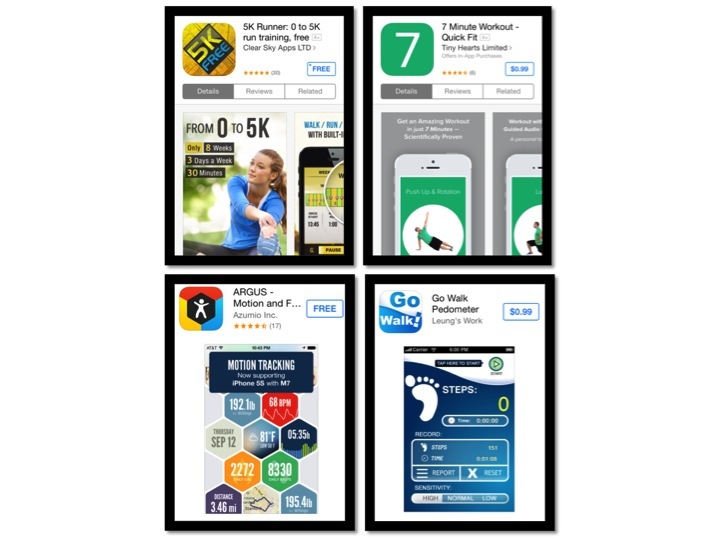
Example of app screenshots.

The search process is outlined in Figure 2. The initial search yielded 2400 apps. After removing duplicate results (n=1282) and ineligible (n=739) apps, descriptive review was conducted on 379 apps (n=206 Apple; n=173 Android). Descriptive results are reported in Table 1. Figure 3 displays examples of screenshots for individual apps from the app stores.

**Table 1:** Descriptive results from primary review (n=379).

Category		n (%)
**Evidence base**		
	Includes public health recommendations for aerobic physical activity quantity	0 (0%)
	Includes public health recommendations for resistance training	7 (1.8%)
**Endorsement**		
	Reference to an affiliated agency	18 (4.7%)
	Academic	5 (27.8%)
	Commercial	13 (72.2%)
**Technological features**		
	Calendar to schedule physical activity	93 (24.5%)
	Reminder to engage in physical activity	45 (11.9%)
	Pairs with a peripheral device	61 (16.1%)
	Capacity for social networking	207 (54.6%)
**Health features**		
	Includes a physical activity target (quantity)	117 (30.9%)
	Records physical data (eg, blood pressure, heart rate, calories, body mass index, limb girths/circumferences)	139 (36.7%)

### Primary Results

The majority of apps were free (237/379, 62.5%), with 142 out of 379 (37.5%) apps charging between CAD $0.99-9.87 to purchase. The most common app price was $0.99. A small portion of apps (18/379, 4.7%) were endorsed by or affiliated with an agency, of which 13 of 18 were commercial (such as corporate apparel and websites) and 5 of 18 were academic (such as research groups and universities).

Descriptive review revealed that no apps (n=0) included evidence-based public health targets for aerobic physical activity, while 7 out of 379 (1.8%) apps included evidence-based public health targets for resistance training. Less than one-third of apps (117/379, 30.9%) included a daily physical activity target, which ranged from pre-set targets (eg, 10,000 steps, 7 minutes, 100 push-ups) through user-defined targets. Methods for measuring physical activity included pedometers (140/379, 36.9%), self-report log (86/379, 22.7%), global positioning system (61/379, 16.1%), accelerometers (44/379, 11.6%), calories expended (89/379, 23.5%), distance (97/379, 25.6%), time (92/379, 24.3%), speed (23/379, 6.1%), and metabolic equivalents (2/379, 0.5%). A combination of these methods was present in nearly half of apps (181/379, 47.8%). In addition to physical activity, 139 out of 379 (36.7%) apps recorded other health data, including calories consumed/expended (52/379, 13.7%), heart rate (38/379, 10.0%), body weight (20/379, 5.3%), body mass index (19/379, 5.0%), limb girths/circumferences (10, 2.6%), and blood pressure (7/379, 1.8%). Similar to measuring physical activity, it was more common for apps to measure a combination of these health parameters (56/139, 40.3%) than a single additional health parameter.

Technological features of interest included capacity for social networking, scheduling features for planning physical activity, reminders to engage in physical activity, and capacity to pair with a peripheral device. Half of apps (207/379, 54.6%) had a social networking capacity; 93 out of 379 (24.5%) included a scheduling feature; 45 out of 379 (11.9%) included a reminder feature; and 61 out of 379 (16.1%) apps paired with a peripheral device, which included a proprietary device like step counters, or associated health devices like weigh scales, heart rate monitors, blood pressure monitors, and glucometers.

### Secondary Results

Detailed review was conducted on a subset (n=26) of apps: the 6 from the initial search that were endorsed by a non-commercial agency (n=5 Apple, n=1 Android); 10 randomly selected from top user ratings (n=5 per platform); and 10 randomly selected from the remainder of the sample (n=5 per platform). In addition to information available from the description page, downloading apps for detailed review revealed that 1 of 26 (3%) apps included reference to public health guidelines for aerobic physical activity, and that 2 of 26 (7%) apps offered features such as social networking, tracking of multiple health parameters, and ideas for physical activity programming available for additional costs (ie, purchase via subscription).

App developers from the subset of apps were contacted, of which 6 of 26 (23%) of developers responded. The physical activity content of 3 apps (coincidentally, 1 from each category selected for detailed review) was inspired by lay and peer-reviewed reports of high-intensity interval training circuits [27,28]; 1 app was also inspired by the physical training manuals for the United States army [29]. One app was inspired by a corporate report on health benefits of physical activity [30]. Two apps were inspired by personal expertise.

## Discussion

### Evidence-Based Content

The present study explored the presence of evidence-based content among physical activity apps marketed through iTunes and Google Play mobile app stores. Previous research examined evidence-based features of smoking cessation, weight loss, including pediatric obesity management, and diabetes management apps, and it was found that the majority of apps do not adhere to evidence-informed practices [21,24,31]. This research identified limitations in analyzing apps for evidence-based content. Review based solely on the description page may bias findings toward marketable features with limited explanation of evidence-based content [22,24]. Therefore, it has been suggested that downloading apps for detailed review may provide more robust data [22,24]. The current study combined both approaches by collecting data from the description page of 379 apps, and subsequently downloading a selected subset of apps based on a priori criteria for further review. Additionally, app developers were contacted to determine evidence-informed content. The combination of approaches for data collection may contribute to a more robust understanding of physical activity apps. Therefore, future research may wish to consider downloading apps for content review as well as communicating with app developers for comprehensive review.

Our primary results demonstrated that no apps included public health recommendations for aerobic physical activity (ie, 150 minutes of moderate- to vigorous-intensity physical activity, in bouts ≥10 minutes). However, detailed review of the subset of apps revealed that one app included reference to evidence-based recommendations for physical activity to inform users, and 4 apps were inspired by various documents about health benefits of physical activity. Additionally, a small proportion (2%) of available apps incorporated whole body strength training of major muscle groups in conjunction with aerobic intervals. The findings suggest limited use of evidence informed practices among physical activity apps. In order to assist users in selecting an app for health promotion, app developers may wish to reference the evidence-based content (eg, public health guidelines for physical activity) on the app’s description page.

### App Features

Descriptive review revealed a broad array of features available within apps, including pairing with peripheral devices to measure markers of health other than physical activity (eg, heart rate, blood pressure, blood glucose). Mobile health devices (such as peripheral devices to measure health markers) can be used to assist both clinicians and clients with evidence-based self-management care and chronic disease prevention [16]. Previous interventions combining mobile health devices and exercise prescription have demonstrated beneficial effects on clinical markers of cardiovascular health [20,32]. Therefore, the ability to connect with additional health devices may be of clinical benefit.

In addition to pairing with peripheral health devices, smartphones allow users to easily share information. Sharing information in a collaborative nature, such as through social media outlets, holds potential for engaging clients in prescribed health behaviors [15,33]. In turn, this may further enable clients and enhance treatment outcomes. Therefore, physical activity apps that include social features such as linking with other users, sharing information, as well as connecting with and building new social networks holds potential for engaging users in planned health behaviors such as physical activity. The present investigation demonstrated that approximately half (54.6%) of physical activity apps included a feature to allow for social networking, such as posting a workout, connecting with other users, and sharing physical activity information with other users. Clinical utility of physical activity apps may be enhanced through inclusion of social networking features.

### Implications for Clinical Use

Direct-access health practitioners, such as primary care physicians, may be the initial health service access point for clients. Therefore, these health practitioners are perhaps ideal clinicians for prescribing physical activity behaviors to reduce economic and disease burden among clients. It has been suggested that a health message delivered in the primary care setting can be an important catalyst for change, in part by representing the value a health practitioner places on health behaviors such as physical activity [11,12,34]. Moreover, physical activity prescription through primary care can significantly increase self-reported physical activity levels, and positively impact cardiorespiratory fitness [14]. These health outcomes from prescribing physical activity may benefit public health, in part by reducing economic burden of unhealthy physical activity behaviors. However, regular visits for healthy clients are generally only one time per year and adherence to physical activity prescription tends to decrease over time. An evidence-based mobile health app could be one tool/strategy to assist clients with engaging in healthy physical activity behaviors during the gap between appointments. For example, recent evidence supports the use of an app through primary care to promote walking [35]. The ability to assess an app’s adherence with evidence-informed practices is of use for both clinicians and clients [24]. Unfortunately, we are unaware of efficacy studies for publicly available physical activity apps available on smartphones to promote physical activity behaviors prescribed by clinicians.

### Limitations

We are aware of limitations in our assessment of physical activity apps. Features available via additional subscription were not included in the present evaluation. For example, apps that required download of additional features for cost, or subscription/membership once the app has been purchased were not considered in the descriptive analysis. Moreover, no rating was made on reliability or validity of physical activity measures within the apps, such as accuracy of step counters, body mass index calculator, heart rate monitors, etc. Some apps were specific to a proprietary device (eg, app that pairs with a brand-specific step counter). These apps were included in the present review; however, the cost for purchasing the proprietary device was not considered in the analysis of cost to download the app.

Moreover, the present review was limited to mobile apps on two of the leading platforms (ie, Apple, Android). Future research may benefit from including additional platforms. Additionally, Google Play search results generate a list of apps available on multiple devices (eg, computer, tablet, smartphone) while the iTunes mobile app store was limited to apps available on smartphone (ie, iPhone) only. Of particular interest for future investigation may be seeking apps available on various devices, including those that do not require a mobile (smart)phone subscription or monthly cellular data plan. This may enhance the applicability of results to allow for use by clients who do not have mobile data plans.

Previously it has been reported that physical activity apps lack sufficient inclusion of health behavior change theories [25]. While the present study is limited in assessing apps for inclusion of evidence-based physical activity guidelines, additional research may wish to explore adherence of physical activity apps to change user’s activity behaviors. Previous studies have explored the availability of evidence-based mobile phone apps to change health-related behaviors such as smoking, chronic disease management, exercise, and obesity prevention [21-24,31]. More research is warranted that explores inclusion of techniques grounded in behavioral change theories to promote adoption of healthy physical activity behaviors (eg, motivational interviewing, transtheoretical model, social cognitive theory, health belief model, theory of planned behavior, behavior modification, social learning theory, and theory of reasoned action [14,36]).

Currently, there is no standardized tool to assess clinical features or evidence-based content of mobile apps. Future research may benefit from development of a tool to systematically assess apps. In the absence of such tool, the present review is limited to assessing evidence-based content in relation to established public health guidelines for physical activity.

The present investigation describes the current availability of physical activity apps. The rate of technological development far outpaces the research process. As such, findings should be considered in the context of selecting an app for use in practice or for developing an evidence-based physical activity app. For this purpose, reference to apps by proprietary name has been intentionally limited, as it was not the goal of this paper to recommend a specific app for use in clinical practice.

### Conclusion

The present review demonstrates a shortage of evidence-based physical activity apps. This gap underscores the need for development of evidence-informed mobile apps. Results highlight the opportunity to develop evidence-informed mobile apps that can be used clinically to enhance health outcomes. Additionally, social integration features (eg, sharing and connecting with others) as well as technological features (eg, pairing with peripheral health devices) may offer the greatest potential to enhance health outcomes among clients prescribed healthy physical activity behaviors.
